# Characterization of Electricity Generated by Soil in Microbial Fuel Cells and the Isolation of Soil Source Exoelectrogenic Bacteria

**DOI:** 10.3389/fmicb.2016.01776

**Published:** 2016-11-08

**Authors:** Yun-Bin Jiang, Wen-Hui Zhong, Cheng Han, Huan Deng

**Affiliations:** ^1^Jiangsu Provincial Key Laboratory of Materials Cycling and Pollution Control, School of Geography Science, Nanjing Normal UniversityNanjing, China; ^2^Jiangsu Center for Collaborative Innovation in Geographical Information Resource Development and ApplicationNanjing, China; ^3^School of Environment, Nanjing Normal UniversityNanjing, China

**Keywords:** Fe(III)-reducing bacteria, Illumina pyrosequencing, *Clostridiaceae*, polarization curve, soil property

## Abstract

Soil has been used to generate electrical power in microbial fuel cells (MFCs) and exhibited several potential applications. This study aimed to reveal the effect of soil properties on the generated electricity and the diversity of soil source exoelectrogenic bacteria. Seven soil samples were collected across China and packed into air-cathode MFCs to generate electricity over a 270 days period. The Fe(III)-reducing bacteria in soil were enriched and sequenced by Illumina pyrosequencing. Culturable strains of Fe(III)-reducing bacteria were isolated and identified phylogenetically. Their exoelectrogenic ability was evaluated by polarization measurement. The results showed that soils with higher organic carbon (OC) content but lower soil pH generated higher peak voltage and charge. The sequencing of Fe(III)-reducing bacteria showed that *Clostridia* were dominant in all soil samples. At the family level, *Clostridiales* Family XI incertae sedis were dominant in soils with lower OC content but higher pH (>8), while *Clostridiaceae, Lachnospiraceae*, and *Planococcaceae* were dominant in soils with higher OC content but lower pH. The isolated culturable strains were allied phylogenetically to 15 different species, of which 11 were *Clostridium*. The others were *Robinsoniella peoriensis, Hydrogenoanaerobacterium saccharovorans, Eubacterium contortum*, and *Oscillibacter ruminantium*. The maximum power density generated by the isolates in the MFCs ranged from 16.4 to 28.6 mW m^-2^. We concluded that soil OC content had the most important effect on power generation and that the *Clostridiaceae* were the dominant exoelectrogenic bacterial group in soil. This study might lead to the discovery of more soil source exoelectrogenic bacteria species.

## Introduction

Soil can be used to generate electrical power in microbial fuel cells (MFCs), which convert chemical energy from soil organic compounds into electricity via catalysis by soil source exoelectrogenic microorganisms. The process of soil power generation has several potential applications. Firstly, the pollutant toxicity and soil microbial activity could be monitored by the generated electrical signals of the MFCs, such as peak voltage, quantity of electrons and start-up time (Deng et al., 2014, 2015; Jiang et al., 2015). Secondly, the use of MFCs would lead to the elimination of soil pollutants including phenol, petrol and oil (Huang et al., 2011; Wang et al., 2012). Thirdly, the operation of MFCs mitigates methane emissions from paddy soil and sediment (Arends et al., 2014). MFCs do not need energy input, instead, a small amount of electrical power is generated. Therefore, MFCs are considered a sustainable technology. The performance of these MFCs is largely related to the magnitude of electrical current generated by the exoelectrogenic bacteria in soil. However, little is known about the character of power generation and the diversity of exoelectrogenic bacteria in different soils.

To date, around 50 bacteria belonging to three phyla *Proteobacteria, Firmicutes*, and *Acidobacteria* have been identified as exoelectrogenic (Zhi et al., 2014). Almost all the exoelectrogenic bacteria strains were isolated from wastewater, sediments of lakes and marine environments, rather than from soil. There is a lack of functional gene markers for exoelectrogenic bacteria; therefore, the main methods used to detect the composition of exoelectrogenic bacteria are isolation of pure cultured bacterial strains or sequence alignment of bacterial 16S rRNA genes with those of known exoelectrogenic bacteria (Song et al., 2012). Most of the evidence about the composition of exoelectrogenic bacteria in soil has been obtained using the “sequence alignment” method (Ishii et al., 2008; Ringelberg et al., 2011). However, novel exoelectrogenic bacteria would be excluded if their sequences were not identical to the identified strains. High throughput DNA pyrosequencing allowed the estimation that one gram of soil contains 1000s of bacterial species (Roesch et al., 2007). Therefore, it is necessary to isolate and identify more exoelectrogenic bacteria strains from soil.

Exoelectrogenic bacteria generally possess the ability to reduce Fe(III), and most Fe(III)-reducing bacteria are exoelectrogenic. However, some exoelectrogenic bacteria do not use Fe(III) as the sole acceptor. For example, *Calditerrivibrio nitroreducens* reduces nitrate rather than Fe(III) (Fu et al., 2013), *Desulfobulbus propionicus* reduces both sulfate and Fe(III) (Holmes et al., 2004), and some Fe(III)-reducing bacteria do not possess the ability to generate electrical current in MFCs, such as *Pelobacter carbinolicus* (Richter et al., 2007). As a result, the composition of Fe(III)-reducing bacteria largely represents the exoelectrogenic bacteria in soil (Lovley, 2006).

Soil physiochemical properties affect microbial diversity and activity (Kuramae et al., 2012), and could have major effects on exoelectrogenic microorganisms in soil (Dunaj et al., 2012). We hypothesized that the diversity of Fe(III)-reducing bacteria and exoelectrogenic bacteria isolates, together with the generated electrical power, would vary between different soils. To test our hypothesis, we collected seven soil samples with different physicochemical properties from Northern to Southern China and packed them into MFCs to generate power. Meanwhile, Fe(III)-reducing bacteria from the seven soil samples were sequenced using the Illumina pyrosequencing system, which can sequence millions of amplicons derived from the dominant species and rare species with high sequence quality (Degnan and Ochman, 2012). In addition culturable Fe(III)-reducing bacteria were isolated and subjected to taxonomic analysis, and were inoculated into MFCs to determine their exoelectrogenic activities. Redundancy analysis was conducted to reveal the relationship between Fe(III)-reducing bacteria, soil physiochemical properties and power generation of soil in MFCs. We aimed to (1) understand the soil properties that had strong effects on the generated electricity and the diversity of Fe(III)-reducing bacteria; and (2) isolate exoelectrogenic bacteria from different soils.

## Materials and Methods

### Soil Sampling

Soil samples were collected from seven sites, which were located in the Inner Mongolia Autonomous Region (IM), Hebei Province (HB), Henan Province (HN), Jiangsu Province (JS), Jiangxi Province (JX), Fujian Province (FJ), and Guangxi Zhuang Autonomous Region (GX), respectively. The location information of the sampling sites is shown in Supplementary Table S1. Each site was planted with one dominant vegetation type. In each site, surface soil samples (0–20 cm) from three randomly selected plots (0.5 m × 0.5 m) were collected and mixed to represent a site, after removing the surface litter. After the soil samples were sieved and passed through a 2 mm diameter mesh, they were stored at 4°C for less than 2 weeks before a series of experiments, including Fe(III)-reducing bacteria enrichment, MFCs operation and soil property measurement.

### Soil Property Measurements

Each of the seven soil samples was divided into three aliquots as replicates for MFCs operation. Before the operation, soil physiochemical properties of each aliquot were analyzed using routine methods (Page et al., 1982). Briefly, soil texture was determined by the sieve and pipette method. Soil maximum water holding capacity (MWHC) was determined by the difference between dry and soaked soil weights. Soil pH was measured at 1:2.5 (soil:water) and soil electrical conductivity (EC) at 1:5 (soil:water). Soil cation exchange capacity (CEC) was analyzed by the compulsive exchange method. Soil organic carbon (OC) was determined by K_2_CrO_4_ oxidation; total nitrogen (TN) by Kjeldahl digestion; and total phosphorus (TP) by colorimetry following NaOH digestion. Dissolved organic carbon (DOC; extracted by 0.5 M K_2_SO_4_) and humic carbon (HC; extracted by 0.1 M Na_4_P_2_O_7_ and 0.1 M NaOH) were measured using a TOC analyzer (TOC-L, Shimadzu, Kyoto, Japan). Soil total dissolved iron (DFe_T_) was extracted by 0.2 M H_2_C_2_O_4_-(NH_4_)_2_C_2_O_4_ (McKeague and Day, 1966) and measured by flame atomic absorption spectroscopy (AA240, Agilent Technologies, Santa Clara, CA, USA). Soil microbial biomass carbon (MBC) was measured by the fumigation-extraction method (Vance et al., 1987).

### MFCs Setup and Operation

A picture and schematic diagram of soil MFCs are shown in Supplementary Figure S1. Twenty-one air-cathode MFC reactors were built in beakers with a 6 cm diameter and 13.5 cm height. Square carbon felt (Haoshi, Lanzhou, China) and platinized carbon paper (Hesen, Shanghai, China) were used as the anode and cathode, respectively, with the same area of 9 cm^2^ (side length 3 cm). In each reactor, the anode was embedded with 250 g soil (dry weight) and the cathode was placed on the soil surface. Deionized water was gently poured into the reactor to keep soil flooded. The two electrodes were connected to an external circuit with a resistance of 1000 Ω using titanium wire. The MFCs were operated in triplicate at a constant 30°C in an incubator. Voltage data generated by the MFCs were recorded every 10 min using a data acquisition module. Deionized water was added every 24 h to compensate for water evaporation and maintain the initial state. After 270 days of MFCs operation, flooded soil in each MFC was air dried under open circuit conditions at 30°C and sieved through a 2 mm diameter mesh to measure the soil properties using the same methods as detailed in the previous section. To confirm that the voltage originated from microbial processes rather than chemical reactions, another seven control MFCs were operated under the same conditions but with chloroform fumigation-sterilized soil (Deng et al., 2015; Jiang et al., 2015).

### Enrichment of Fe(III)-Reducing Bacteria

The enrichment of Fe(III)-reducing bacteria in the soil samples was conducted under anaerobic conditions (10% CO_2_, 10% H_2_, and 80% N_2_) in an anaerobic workstation (MiniMacs, Don Whitley Scientific, Shipley, UK). For each soil sample, 2.0 g soil (dry weight) was inoculated into 100 mL basal medium. One liter of basal medium (BM) contained 8 g peptone, 1 g yeast extract, 0.12 g NH_4_Cl, 16 g sodium acetate, 5 g NaCl, 1.2 g K_2_HPO_4_, 1 g cysteine hydrochloride, 1 mg resazurin, 5 mL mineral, and 5 mL vitamin solutions (Sigma-Aldrich, Co., St. Louis, MO, USA) (Lovley and Phillips, 1988). The electron acceptor was 25 mM ferric citrate. The medium was boiled with N_2_ for 20 min and then autoclaved in sealed bottles. The pH of the autoclaved medium was 6.7. The inoculated medium was incubated at 30°C for 7 days under dark conditions. The enrichment procedure was repeated three times (Kim et al., 2005).

### High-Throughput Pyrosequencing

Five-milliliters of the enrichment product were collected after the anaerobic culture and centrifuged at 14,000 × *g* for 10 min. The genomic DNA was immediately extracted from the precipitates using a Fast DNA SPIN kit for soil (BIO101, MP Biomedicals, Carlsbad, CA, USA) following the manufacturer’s instructions. The purity and the quantity of the extracted DNA were determined using a nanodrop UV-Vis spectrophotometer (ND-1000, NanoDrop, Wilmington, DE, USA) at 230, 260, and 280 nm.

The bacterial 16S rRNA genes of the seven enrichments were amplified using universal primers 515F (5′-GTG CCA GCM GCC GCG GTA A-3′) and 907R (5′-CCG TCA ATT CMT TTR AGT TT-3′) (Weisburg et al., 1991). The PCR reactions were quantified and the products were then purified. At least 24,000 reads were conduct for each sample using the Illumina MiSeq platform (Illumina, San Diego, CA, USA) using 2 bp × 250 bp paired end flow cells and reagent cartridges. The Illumina sequencing data were analyzed by Mothur (Schloss et al., 2009) using the MiSeq standard operating procedure (Kozich et al., 2013). The raw data were deposited in the NCBI Sequence Read Archive database with the accession number SRP071622.

### Isolation and Taxonomic Analysis of Fe(III)-Reducing Bacteria

After the three rounds of 7 days anaerobic incubation, 0.5 ml of the enrichment products were separated by the spread plate method after 10-fold dilution for bacteria isolation using solid BM. The plates were incubated for 5 days under anaerobic condition, after which single black colonies were picked out and inoculated into liquid BM. The procedures were repeated three times to obtain pure cultures. All the experiments were conducted in the MiniMacs anaerobic workstation.

The phylogenetic analysis of the isolates was conducted based on the 16S rRNA gene sequences, which were PCR amplified using the primers 27F (5′-AGA GTT TGA TCM TGG CTC AG-3′) and 1492R (5′-GGT TAC CTT TGT TAC GAC TT-3′) (Suzuki and Giovannoni, 1996). The PCR products were cloned using the Peasy^TM^-T3 Cloning Kit (TransGen, Beijing, China) according to the manufacturer’s recommendations. GeneScript (Nanjing, China) sequenced six clones for each isolate. The vector sequences were removed using DNASTAR Lasergene (version 7.1). The gene sequences of the isolates were subjected to taxonomic assignments using BlastX. The sequences of all the isolates were submitted to GenBank with the accession numbers KT889276–KT889290. One strain each of isolate 1 (CGMCC 1.5212) and isolate 2 (CGMCC 1.5211) were deposited in the China General Microbiological Culture Collection Center.

### Electrochemical Tests of the Isolates

One of the strains phylogenetically related to each species was randomly selected and the exoelectrogenic activity of the strains was characterized by polarization curve measurement using H-type dual chamber MFC reactors (100-I, Fuxiao, Changshu, China). The anode and cathode were both rectangular carbon felt with the same area of 18 cm^2^ (3 cm × 6 cm), connected to an external resistance of 1000 Ω using titanium wire. LB medium, which is favorable for power generation by exoelectrogenic bacteria, was used in the subsequent tests of the isolates (Feng et al., 2014). The anodic chamber and cathodic chamber were filled with 120 mL LB medium and 120 mL potassium ferricyanide [100 mM K_3_Fe(CN)_6_ in 50 mM, pH 7 PBS], respectively. The two chambers were separated by a cation exchange membrane (32S, Qianqiu, Hangzhou, China). The reactors and LB medium were autoclaved before use. Voltage data were recorded every 20 min using a data acquisition module.

The polarization curves of the MFC reactors with pure isolates were measured in the fed-batch mode (Lovley and Phillips, 1988). Briefly, the liquid medium containing isolated cells (200 μL) was inoculated into the anodic chamber. Eighty-milliliters of medium were replaced after the voltage of MFCs reached its peak. The reactors were then operated under open circuit conditions for 4 h and with different loads (50,000, 10,000, 5,000, 1,000, 800, 500, 300, 200, and 100 Ω) for 20 min for each load. All the MFCs were operated at a constant 30°C in an incubator.

### Statistical Analysis

The charge generated from the MFCs, defined as the quantity of the generated electrons, was calculated as previously described (Deng et al., 2015). Current density was calculated from the external load and cell voltage according to Ohm’s law (I = U/R) and normalized to the surface area (m^2^) of the cathodic electrode. The power density was calculated by using P (mW m^-2^) = 10 × U^2^/(R × A), where U (mV) is the recorded voltage, A (m^2^) is the surface area of the anode, and R (Ω) is the external load (Cheng et al., 2006).

Significant differences between means were determined by one-way ANOVA at a level of *P* < 0.05, using the least significance difference (LSD) test. Cluster analysis of the Fe(III)-reducing bacteria community was conducted based on the square Euclidean distance by the between-groups linkage method. Redundancy analysis (RDA) was carried out by Canoco for Windows (version 4.5) between the Fe(III)-reducing bacteria community and environmental or electrical variables, which were selected using the Monte Carlo permutations test (499 permutations). All statistical tests, except for RDA, were performed using SPSS software (version 18.0).

## Results

### Power Generation of Soils in MFCs

**FIGURE 1:**
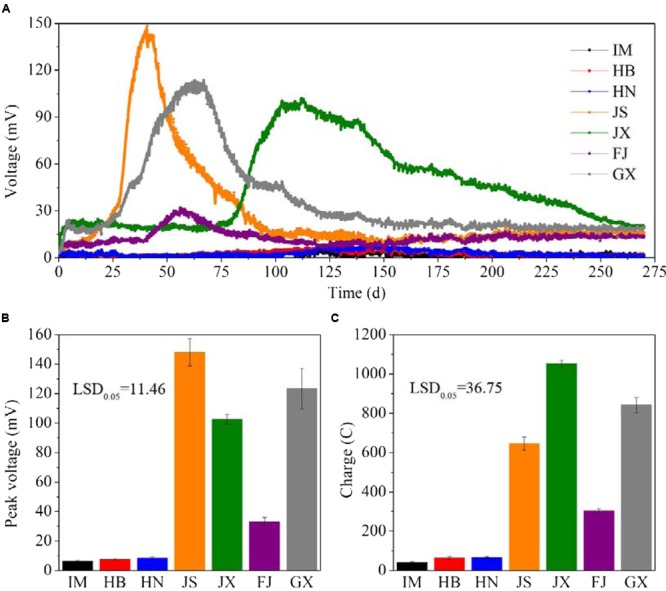
**The V-t curves (A)**, peak voltage **(B)** and charge quantity **(C)** of MFCs constructed using the seven soils. Data are presented as means (*n* = 3) in **(A)** and means with error bars as standard error (*n* = 3) in **(B,C)**. LSD_0.05_ represents the least significant difference at level of *P* < 0.05. IM, HB, HN, JS, JX, FJ, and GX represent the seven sampling sites in Inner Mongolia, Hebei, Henan, Jiangsu, Jiangxi, Fujian, and Guangxi, respectively.

The electricity generation by MFCs comprising the seven soils lasted for 270 days (**Figure 1A**). The voltage curves of the MFCs were characterized by a single peak. The seven soils varied in their peak voltage and also the time from the beginning to the peak. It took about 40 days for JS, 60 days for both FJ and GX, and 100 days for JX to reach to their peak voltages. IM, HB and HN reached their peaks at round 130 days. The peak voltages of JS, GX and JX were 148.1, 123.3, and 102.6 mV, respectively. They were significantly higher (*P* < 0.05) than FJ (32.9 mV), HN (8.4 mV), HB (7.4 mV), and IM (6.2 mV) (**Figure 1B**). After the peak data, the voltage continued to decrease until the end of MFCs operation, when the voltage data of all soils were below 30 mV. During the 270 days of MFCs operation, JX generated the highest charge (1052.4 C), followed by GX (842.2 C), JS (644.7 C), FJ (302.2 C), HN (66.4 C), HB (63.5 C), and IM (41.9 C) (**Figure 1C**). No voltages were detected in any MFCs containing sterilized soil samples, indicating that the electricity generation originated from microbial catalysis rather than chemical reactions.

### Soil Properties before and after MFCs Operation

The soil physicochemical and microbial properties varied between soils (**Table 1**). Before MFCs operation, the CEC, OC, TN, and HC values in soils from JS and JX were significantly higher (*P* < 0.05) than the other soils. JS exhibited the highest MBC, followed by JX and GX. JX also had the highest DFe_T_. Soils from IM, HB and HN exhibited higher soil pH (>8) but lower OC, DOC, HC, and MBC compared with the other soils. Compared with soil physiochemical and microbial properties before MFCs operation, after 270 days MFCs operation, the OC and DOC of IM, HB and HN did not change significantly; however, those of JS, JX, FJ, and GX decreased significantly (*P* < 0.05). The MBC of all the seven soils decreased significantly after MFCs operation.

**Table 1:** Soil physicochemical and microbial properties before and after MFCs operation.

	Soil	pH	OC (g kg^-1^)	TN (g kg^-1^)	TP (g kg^-1^)	DOC(mg kg^-1^)	HC (g kg^-1^)	DFe_T_(mg kg^-1^)	MBC(mg kg^-1^)
Before MFCs operation	IM	8.51 (0.01)	8.31 (1.71)	1.12 (0.02)	0.38 (0.06)	44.02 (6.99)	4.76 (0.05)	381.79 (15.92)	125.39 (18.62)
	HB	8.33 (0.04)	6.38 (0.70)	0.88 (0.06)	1.53 (0.26)	51.26 (2.49)	4.90 (0.33)	123.95 (7.66)	21.41 (5.62)
	HN	8.26 (0.01)	11.18 (3.28)	1.05 (0.03)	1.71 (0.30)	56.73 (7.07)	5.63 (0.19)	133.15 (11.16)	116.52 (15.93)
	JS	7.07 (0.02)	25.41 (2.87)	2.26 (0.35)	0.96 (0.11)	75.47 (6.02)	9.91 (0.16)	401.39 (1.28)	377.96 (38.98)
	JX	4.12 (0.01)	24.83 (2.71)	1.71 (0.04)	0.40 (0.10)	148.88 (11.12)	10.26 (0.34)	547.84 (14.00)	304.55 (22.85)
	FJ	7.96 (0.03)	12.60 (0.51)	1.21 (0.05)	1.85 (0.36)	123.47 (21.62)	5.29 (0.49)	150.87 (5.55)	147.34 (32.98)
	GX	4.04 (0.03)	14.96 (0.62)	1.30 (0.02)	0.27 (0.03)	228.85 (11.25)	6.86 (0.87)	347.03 (11.69)	247.76 (22.06)
After MFCs operation	IM	8.22 (0.02)	8.51 (1.28)	1.22 (0.09)	0.42 (0.16)	39.24 (4.72)	3.05 (0.08)	410.43 (10.61)	26.43 (5.41)
	HB	8.24 (0.08)	7.33 (0.49)	0.86 (0.01)	1.25 (0.22)	44.18 (4.54)	2.05 (0.16)	100.74 (4.47)	37.96 (8.92)
	HN	8.12 (0.03)	9.97 (1.46)	1.08 (0.04)	1.63 (0.04)	48.71 (1.02)	2.75 (0.32)	108.99 (8.29)	38.71 (2.87)
	JS	6.17 (0.02)	14.53 (0.77)	2.23 (0.07)	0.95 (0.18)	46.47 (4.28)	9.85 (0.43)	383.99 (17.57)	78.18 (6.75)
	JX	4.61 (0.03)	14.52 (0.60)	1.59 (0.03)	0.39 (0.03)	77.76 (4.64)	10.52 (0.68)	559.74 (6.74)	90.04 (13.83)
	FJ	8.16 (0.03)	6.03 (1.23)	1.07 (0.07)	1.32 (0.08)	67.89 (11.13)	2.69 (0.66)	128.75 (24.33)	82.78 (17.76)
	GX	4.98 (0.05)	8.06 (0.56)	1.35 (0.03)	0.54 (0.09)	49.99 (1.77)	7.25 (0.33)	351.29 (5.27)	76.07 (5.64)
LSD_0.05_		0.05	2.72	0.55	0.29	14.62	0.72	19.87	31.44

*LSD_*0.05*_ represents the least significant difference at level of *P* < 0.05. Data are presented as means with the standard error in parenthesis. OC, organic carbon; TN, total nitrogen; TP, total phosphorus; DOC, dissolved organic carbon; HC, total humic carbon; DFe_*T*_, total dissolved iron; MBC, microbial biomass carbon.*

### Diversity of Fe(III)-Reducing Bacteria in the Soils

**FIGURE 2:**
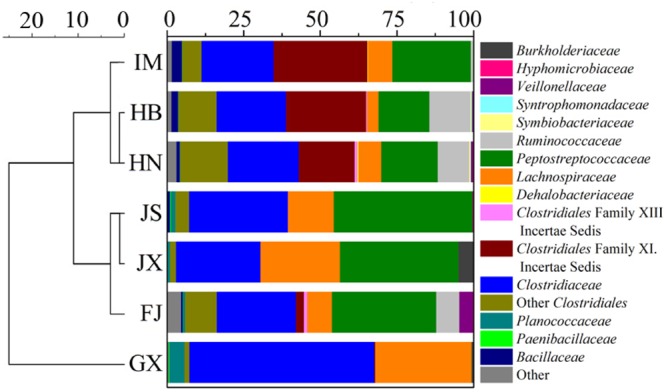
**Taxonomic classification of DNA sequences and cluster analysis of the Fe(III)-reducing bacteria community at the family level in the seven soils.** The Fe(III)-reducing bacteria that comprised less than 0.1% of total composition in all libraries were not included. IM, HB, HN, JS, JX, FJ, and GX represent seven sampling sites in Inner Mongolia, Hebei, Henan, Jiangsu, Jiangxi, Fujian, and Guangxi, respectively.

The sequences of Fe(III)-reducing bacteria in the seven soils were assigned to the known phyla, class and family. Four phyla were observed, with *Firmicutes* being the overwhelmingly dominant phylum (Supplementary Figure S2A). Six classes were detected, including α-, β- and δ-*Proteobacteria, Clostridia, Bacilli*, and *Actinobacteria* (Supplementary Figure S2B). The most abundant class was *Clostridia*, which accounted for over 90% of the total composition in the seven soils. At the family level, the composition of Fe(III)-reducing bacteria was different between soils (**Figure 2**). In IM, HB and HN, *Clostridiales* Family XI. incertae sedis accounted for 30.5, 26.0, and 18.3% of total composition, respectively. However, it was a minor bacterial group in FJ (2.6%), GX (0.3%), JS (<0.1%), and JX (<0.1%). By contrast, the relative abundances of *Lachnospiraceae* in IM (7.9%), HB (3.7%), and HN (7.4%) were lower than those in JS (14.7%), JX (25.7%), and GX (29.9%). GX was dominated by *Clostridiaceae* (57.5%) and *Lachnospiraceae* (31.4%). The relative abundance of *Peptostreptococcaceae* in GX was only 0.1%, which was much lower than that of the other six soils (16.5∼38.5%). *Eubacteriaceae* and *Ocillospiraceae* were detected in FJ, although their relative abundances were less than 0.1%. Cluster analysis of the Fe(III)-reducing bacterial community at the family level revealed that IM, HB, and HN were grouped in one cluster, while JS, JX, and FJ were in another cluster. GX was not grouped with either cluster (**Figure 2**).

### Relationships between Fe(III)-Reducing Bacteria and Soil Properties

**FIGURE 3:**
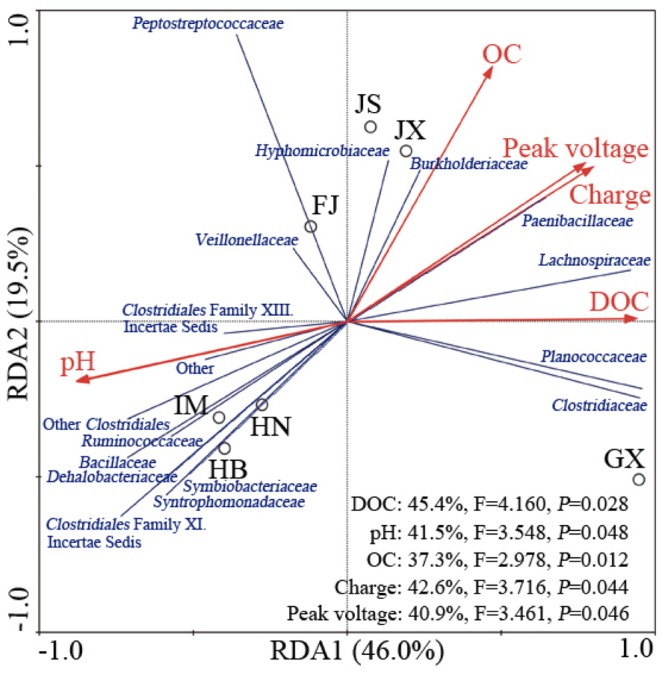
**Redundancy analysis between the Fe(III)-reducing bacteria community at the family level and selected environmental or electrical variables in the seven soils.** Circles, red arrows and blue lines represented community, variables and families, respectively. The Fe(III)-reducing bacteria that comprised less than 0.1% of total composition in each soil were not included. IM, HB, HN, JS, JX, FJ, and GX represent seven sampling sites in Inner Mongolia, Hebei, Henan, Jiangsu, Jiangxi, Fujian, and Guangxi, respectively. The corresponding explained proportions of variability between variables and community are shown in the lower right corner.

Redundancy analysis showed that the first two components (RDA1 and RDA2) together explained 65.5% of the total variation of the Fe(III)-reducing bacteria (**Figure 3**). Along RDA1, IM, HB, and HN were separated from FJ, JS and JX, and GX was separated from the two groups. RDA2 mainly separated the group comprising FJ, JS and JX from the other four soils. The relative abundances of the families *Clostridiales* Family XI. incertae sedis, *Ruminococcaceae, Bacillaceae, Dehalobacteriaceae, Symbiobacteriaceae*, and *Syntrophomonadaceae* were positively related to soil pH and were increased in IM, HB, and HN. The relative abundances of the families *Peptostreptococcaceae, Hyphomicrobiaceae, Burkholderiaceae*, and *Veillonellaceae* were positively related to soil OC and were increased in JS, JX and FJ. The families *Clostridiaceae* and *Planococcaceae* were more abundant in GX, and their relative abundances were positively correlated with soil DOC. Peak voltage and charge, which increased along with RDA1 and RDA2, were positively correlated with the families *Hyphomicrobiaceae, Burkholderiaceae, Paenibacillaceae, Lachnospiraceae, Planococcaceae*, and *Clostridiaceae*.

### Taxonomic and Electrochemical Analysis of Fe(III)-Reducing Bacteria Isolates

**FIGURE 4:**
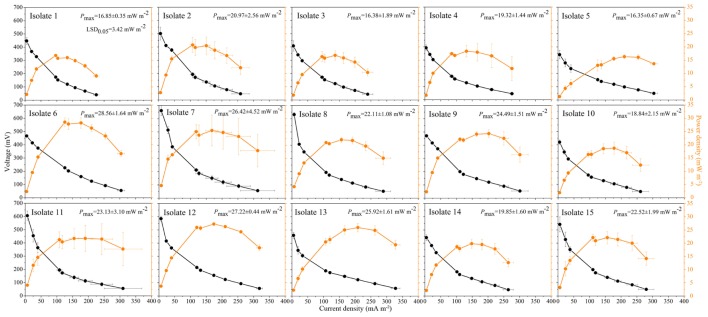
**Polarization curves (black circles) and power density curves (orange circles) from microbial fuel cells using the 15 isolates as the anodic biocatalyst.** Data are presented as means with error bars as standard error (*n* = 3).

Culturable strains isolated from the seven soils were phylogenetically related to 15 different species, of which 11 were *Clostridium* spp. (**Table 2**). Isolate 12, belonging to the *Lachnospiraceae*, was isolated from JS. Isolates 13–15, belonging to *Ruminococcaceae, Eubacteriaceae*, and *Ocillospiracea*, respectively, were isolated from FJ. Polarization and power density curves of the 15 isolates are shown in **Figure 4**. The MFCs catalyzed by the 15 isolates showed an open circuit voltage ranging from 400 to 630 mV. The cell voltage decreased, while the electrical current increased, with decreasing external load. The voltage drops of the polarization curves showed activation losses, ohmic losses and concentration losses. The power density curves of the 15 isolates peaked at the range of 16.4∼28.6 mW m^-2^. Isolate 6, which was related to both *Clostridium amylolyticum* and *Clostridium mesophilum*, exhibited the highest *P*_max_ (28.6 mW m^-2^) of all the isolates.

**Table 2:** Taxonomy based on 16S rRNA genes and sources of Fe(III)-reducing bacteria isolates.

Isolate	Related species	Accession number^a^	Identity	Family	Source
1	*Clostridium sporogenes* *Clostridium botulinum*	CP009225 CP000726	99% 99%	*Clostridiaceae*	IM, HB, HN, JS, JX, FJ, GX
2	*Clostridium bifermentans*	JX267051	99%		IM, HB, HN, JS, JX
3	*Clostridium glycolicum*	KJ722507	99%		HB, FJ, GX
4	*Clostridium irregulare*	EU887817	99%		HN, FJ
5	*Clostridium* sp.	FJ384387	99%		HB, HN
6	*Clostridium amylolyticum*, *Clostridium mesophilum*	NR044386 JN650296	99% 99%		HB
7	*Clostridium beijerinckii*	CP006777	99%		HN
8	*Clostridium venationis*	EU089966	99%		JS
9	*Clostridium celerecrescens*	JN650298	99%		JS
10	*Clostridium subterminale*, *Clostridium thiosulfatireducens*	NR113027 NR042718	99% 99%		FJ
11	*Clostridium sphenoides*, *Clostridium celerecrescens*	LC053840 JN650298	99% 99%		JS, JX, GX
12	*Robinsoniella peoriensis*	AF445283	99%	*Lachnospiraceae*	JS
13	*Hydrogenoanaerobacterium saccharovorans*	NR044425	99%	*Ruminococcaceae*	FJ
14	*Eubacterium contortum*	EU980608	99%	*Eubacteriaceae*	FJ
15	*Oscillibacter ruminantium*	NR118156	99%	*Ocillospiraceae*	FJ

*^a^Nucleotide sequence accession number of the related species in the GenBank database.*

## Discussion

In the present study, we investigated the characters of power generation by seven soils in MFCs over a 270 days period, and revealed that the soil OC content had the most important effect on power generation. We isolated 15 strains of exoelectrogenic bacteria from the seven soil samples, and most of them were related to *Clostridium* spp. These soil source exoelectrogenic bacteria isolates have not been reported before.

Soil from JX, GX, and JS generated higher peak voltages and charges compared with the other soils. This result might be explained by the higher OC content in JX, GX, and JS compared with the other soils. The RDA showed that the peak voltage and charge were positively correlated with the OC and DOC content, but negatively correlated with soil pH. The OCs served as electron donors for the exoelectrogenic bacteria, whose activity increased with the input of OCs (Di Lorenzo et al., 2009). The voltage gradually decreased after the peak voltage, especially for the JX, JS, and GX samples. Soil OC and DOC content in JX, JS, and GX significantly decreased after MFCs operation, compared with before MFCs operation, suggesting that the rapid exhaustion of OCs leads to decreased power output. By contrast, studies have shown that in air cathode MFCs, the optimal pH was between 8 and 10 for exoelectrogenic bacteria to generate power (He et al., 2008). The negative relationships between soil pH and peak voltage and charge should not indicate that acidic soil favors power generation. Therefore, we suggest that the OC and DOC contents might be more important drivers of power generation than soil pH.

Our results demonstrated that *Firmicutes* and *Clostridia* dominated the phylum and class level of Fe(III)-reducing bacteria, respectively. A previous study also found that in paddy soil, *Firmicutes* and *Clostridia* accounted for 80 and 52% of Fe(III)-reducing bacteria, respectively (Li et al., 2011). *Clostridiales* Family XI. incertae sedis was abundant in IM, HB, and HN. However, it was a minor group in FJ and was undetected in JS, JX, and GX. Similarly, *Peptostreptococcaceae*, which was undetected in GX, was abundant in the other six soil samples. A possible reason was that the two bacterial groups were sensitive to decreased soil pH. GX had the lowest pH, which might cause stress to the bacteria, leading to the lowest diversity of Fe(III)-reducing bacteria. Nevertheless, GX generated a relatively high voltage and charge. The RDA analysis suggested that three dominant bacterial groups, *Clostridiaceae, Lachnospiraceae* and *Planococcaceae*, prefer low pH and high OC content in GX, and might be important for power generation. In addition, *Clostridiaceae, Lachnospiraceae* and *Peptostreptococcaceae*, which belong to *Clostridia*, were abundant in most tested soils and their levels were positively related to peak voltage and charge in the RDA analysis, indicating that the *Clostridia* class might play an important role in power generation in soils.

All 15 isolates of Fe(III)-reducing bacteria were confirmed to have exoelectrogenic ability by the polarization test, which is one of the most widely used techniques to determine the bioelectrochemical activity of exoelectrogenic bacteria and to test MFCs performance (Puig et al., 2010; Luo et al., 2015). Most exoelectrogenic bacteria isolates are Gram-negative and belong to the phylum *Proteobacteria* (Zhi et al., 2014). The first Gram-positive bacterium demonstrated to produce electricity in MFCs was *Clostridium butyricum* EG3 (Park et al., 2001). Our results demonstrated that *Clostridium* was the dominant exoelectrogenic bacterial group in the studied soil samples. Isolates 1 to 11 were related genetically to *Clostridium* species. It was reported that *Clostridium butyricum* had membrane-bound cytochromes which carried out the direct electron transfer (Park et al., 2001). Species of the same genus might share the same mechanism of electron transfer. *Hydrogenoanaerobacterium saccharovorans*, which was closely related to isolate 13, produces H_2_ during growth (Song and Dong, 2009). H_2_ /H^+^ could mediate the electron transfer from exoelectrogenic bacteria to the electrode (Rosenbaum et al., 2005). *Robinsoniella peoriensis, Eubacterium contortum* and *Oscillibacter ruminantium*, which were genetically related to isolates 12, 14, and 15, respectively, have not been reported as exoelectrogenic bacteria before, and their electron transfer mechanisms remain unknown.

In our study, acetate was used as a carbon substrate in the enrichment and isolation of Fe(III)-reducing bacteria. Both acetate and glucose are the most easy-to-degrade substrates for exoelectrogenic bacteria. However, acetate exhibits higher coulombic efficiency than glucose and thus it has become the most widely applied substrate (Pham et al., 2003; Zuo et al., 2008; Fu et al., 2013). Nevertheless, some exoelectrogenic bacteria do not utilize acetate as an optimal carbon substrate (Xu and Liu, 2011; Feng et al., 2014; Luo et al., 2015), and some cannot metabolize acetate at all. For example, *Shewanella oneidensis* oxidizes lactate rather than acetate under anaerobic conditions (Lovley et al., 1993). In addition, some exoelectrogenic bacteria are unable to reduce Fe(III) (Fu et al., 2013). We suggest that more exoelectrogenic bacterial strains should be isolated from soils using more kinds of donors and acceptors.

## Author Contributions

HD conceived the idea. Y-BJ and W-HZ conducted all the experiments. CH conducted the data analysis. Y-BJ wrote the first draft and HD finalized the manuscript with assistance from all co-authors.

## Conflict of Interest Statement

The authors declare that the research was conducted in the absence of any commercial or financial relationships that could be construed as a potential conflict of interest.
