# Supplementary material for: Characterization of Electricity Generated by Soil in Microbial Fuel Cells and the Isolation of Soil Source Exoelectrogenic Bacteria
Source: Front Microbiol. 2016 Nov 8;7:1776. doi: 10.3389/fmicb.2016.01776 (PMC5099896; doi:10.3389/fmicb.2016.01776)
Supplement: Supplementary file 1 [file Data_Sheet_1.doc]

**Supplemental Material for**

Characterization of Electricity Generated by Soil in Microbial Fuel Cells and the Isolation of Soil Source Exoelectrogenic Bacteria

Yun-Bin Jiang^1, 2^, Wen-Hui Zhong^1, 2^, Cheng Han^1, 2^, Huan Deng^1, 2, 3,*^

^1^Jiangsu Provincial Key Laboratory of Materials Cycling and Pollution Control, School of Geography Science, Nanjing Normal University, Nanjing 210023, China

^2^Jiangsu Center for Collaborative Innovation in Geographical Information Resource Development and Application, Nanjing 210023, China

^3^School of Environment, Nanjing Normal University, Nanjing 210023, China

^*^Corresponding author: Huan Deng

E-mail address: [hdeng@njnu.edu.cn](mailto:hdeng@njnu.edu.cn)

This file includes:

Supplementary Table S1 and Fig. S1-S2

**Table S1** Sampling site location, sample texture and physical properties of the seven soils.

| Soil | Location | Average annual temperature (°C) | Average annual precipitation (mm) | Dominant vegetation type^a^ | Soil Texture^b^ | Sand  (%) | Clay  (%) | WHC  (%) | EC  (μS cm^-1^) | CEC  (cmol kg^-1^) |
| --- | --- | --- | --- | --- | --- | --- | --- | --- | --- | --- |
| IM | N40°44′50″  E111°38′57″ | 5.8 | 378 | *Salix matsudana* | Sandy clay loam | 56.42(1.03) | 22.24(0.76) | 28.84(1.87) | 99.33(2.73) | 14.55(0.26) |
| HB | N39°31′47″  E116°41′40″ | 12.0 | 550 | *Sophora japonica* | Medium loam | 46.43(1.97) | 18.78(0.61) | 36.73(5.48) | 135.4(3.56) | 7.82(2.73) |
| HN | N34°48′27″  E114°20′15″ | 14.0 | 670 | *Sophora japonica* | Silty loam | 21.80(3.60) | 25.54(1.08) | 38.68(2.63) | 149.0(4.49) | 8.55(0.30) |
| JS | N32°03′11″  E118°49′39″ | 15.4 | 1106 | *Platanus orientalis* | Silty clay | 12.02(1.14) | 39.06(0.70) | 59.91(1.28) | 105.4(2.18) | 23.95(0.26) |
| JX | N28°15′20″  E116°55′30″ | 17.8 | 1785 | *Lespedeza bicolor* | Clay | 19.04(1.15) | 54.68(2.60) | 57.79(5.58) | 78.43(1.14) | 18.58(0.64) |
| FJ | N24°34′18″  E118°06′08″ | 21.0 | 1200 | *Ficus microcarpa* | Loamy sand | 77.32(4.11) | 10.39(3.60) | 27.53(2.53) | 278.7(8.74) | 5.40(0.79) |
| GX | N24°16′35″  E109°24′33″ | 20.5 | 1400 | *Ficus microcarpa* | Silty clay loam | 17.89(0.84) | 37.17(0.39) | 71.87(3.56) | 93.80(3.14) | 13.73(0.34) |

^a^ The planting ages of these vegetation types in the sampling sites are all over 20 years. ^b^ Soil texture was classified according to US soil taxonomy. Data were presented as means with standard error in parenthesis. WHC: maximum water holding capacity, EC: electrical conductivity, CEC: cation exchange capacity. IM, HB, HN, JS, JX, FJ and GX represent seven sampling sites in Inner Mongolia, Hebei, Henan, Jiangsu, Jiangxi, Fujian and Guangxi.


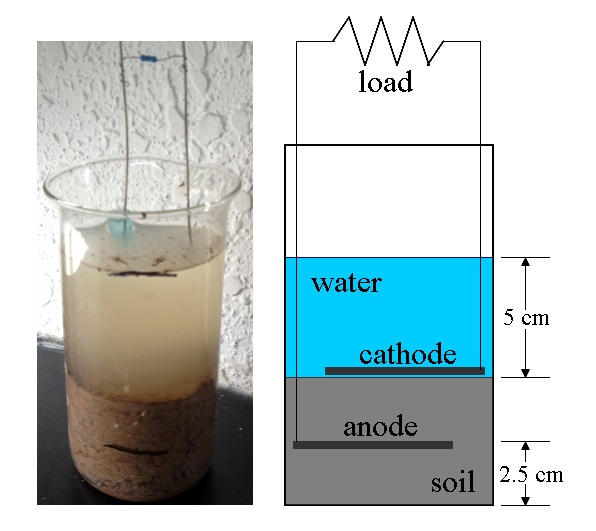


**Fig. S1** Picture and schematic diagram of MFC constructed using soil for bioelectricity generation.


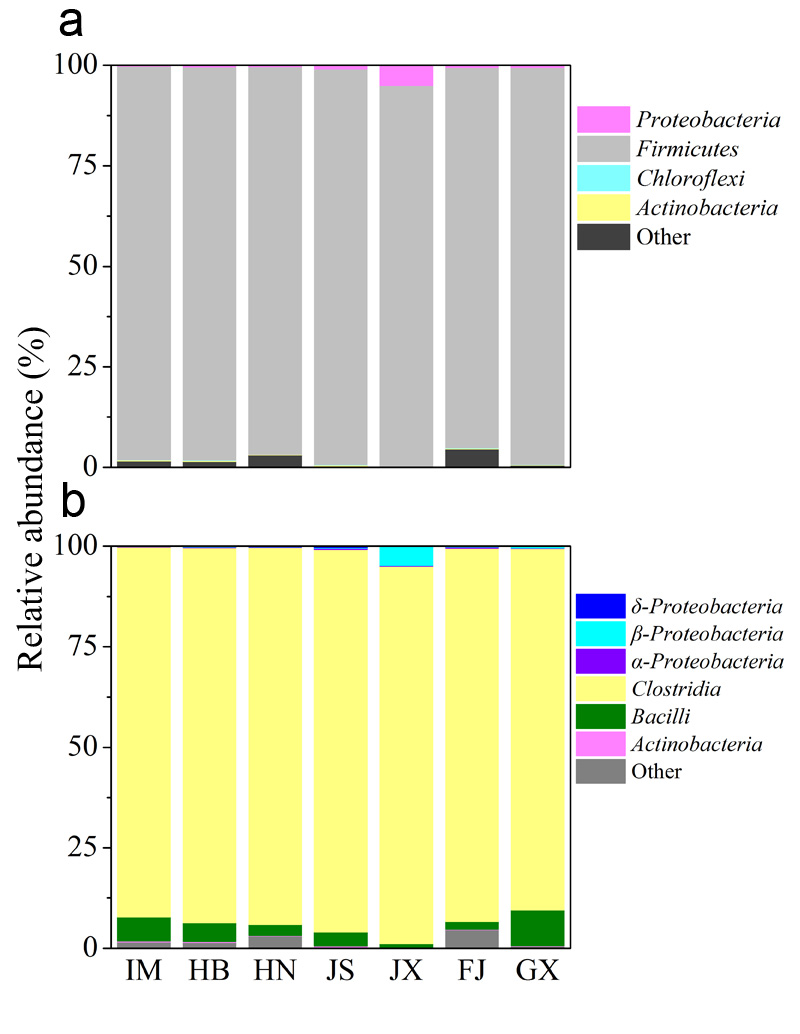


Fig. S2 Taxonomic classification of DNA sequences from Fe(III)-reducing bacterial communities of seven soils at the phylum level (a), the class level (b). The ones that comprised less than 0.1% of total composition in all libraries were not included. IM, HB, HN, JS, JX, FJ and GX represent seven sampling sites in Inner Mongolia, Hebei, Henan, Jiangsu, Jiangxi, Fujian and Guangxi.
